# A comparison of Zika and dengue outbreaks using national surveillance data in the Dominican Republic

**DOI:** 10.1371/journal.pntd.0006876

**Published:** 2018-11-05

**Authors:** Leigh R. Bowman, Joacim Rocklöv, Axel Kroeger, Piero Olliaro, Ronald Skewes

**Affiliations:** 1 Department of Public Health and Clinical Medicine, Unit of Epidemiology and Global Health, Umeå University, Umeå, Sweden; 2 UNICEF/UNDP/World Bank/WHO Special Programme for Research and Training in Tropical Diseases (TDR), Geneva, Switzerland; 3 Department of Public Health, Ministry of Health, Santo Domingo, Dominican Republic; University of Queensland, AUSTRALIA

## Abstract

**Background:**

*Aedes*-borne arboviruses continue to precipitate epidemics worldwide. In Dominican Republic, the appearance of Zika virus cases that closely followed a large dengue epidemic provided an opportunity to study the different transmission drivers behind these two flaviviruses. Retrospective datasets were used to collect information on the populations at risk and descriptive statistics were used to describe the outbreaks on a national scale.

**Methodology/ Principal findings:**

Expectedly, box plots showed that 75% of dengue was reported in those aged <20 years while Zika infections were more widely dispersed among the population. Dengue attack rates were marginally higher among males at 25.9 per 10,000 population vs. 21.5 per 10,000 population for females. Zika infections appeared to be highly clustered among females (73.8% (95% CI 72.6%, 75.0%; p<0.05)); age-adjusted Zika attack rates among females were 7.64 per 10,000 population compared with 2.72 per 10,000 population among males. R0 calculations stratified by sex also showed a significantly higher metric among females: 1.84 (1.82, 1.87; p<0.05) when compared to males at 1.72 (1.69, 1.75; p<0.05). However, GBS attack rates stratified by sex revealed slightly higher risk in males vs. females, at 0.62 and 0.57 per 10,000 population respectively.

**Conclusions/ Significance:**

Evidence suggests little impact of existing dengue immunity on reported attack rates of Zika at the population level. Confounding of R0 and incident risk calculations by sex-specific over-reporting can alter the reliability of epidemiological metrics, which could be addressed using associated proxy syndromes or conditions to explore seemingly sex-skewed incidence. The findings indicate that community awareness campaigns, through influencing short-term health seeking behaviour, remain the most plausible mechanism behind increased reporting among women of reproductive age, although biological susceptibility cannot yet be ruled out. Media campaigns and screening are therefore recommended for women of reproductive age during Zika outbreaks. Future research should focus on clinical Zika outcomes among dengue seropositive individuals.

## Author summary

During the years 2013–2016, worldwide Zika epidemics spread quickly throughout immune-naïve human populations. In 2015–2016, the Dominican Republic was struck by an epidemic of dengue followed by an outbreak of Zika. The Zika epidemic closely followed the tail of the dengue epidemic and provided an opportunity to study the dynamics of Zika and dengue transmission at the population level over a similar timeframe. The findings showed that while dengue cases were marginally higher among males, Zika cases were much higher among females and in particular among sexually active age groups. This might indicate that 1) prior dengue infections (and hence antibody cross-reactivity) in these populations had no material impact on the likelihood of reporting Zika illness 2) over-reporting in females was due to health campaigning that targeted females or 3) there was a biological cause for increased susceptibility among women. These possible causes are not mutually exclusive but it is most likely that the clustering of cases among women was the result of health-seeking behaviour caused by health campaigns and media coverage of Zika at the time. If so, media campaigns can be an effective strategy for encouraging health-seeking behaviour among target demographics.

## Introduction

Arboviruses are responsible for increasing worldwide morbidity and mortality at an alarming rate. While new technologies are emerging to reduce transmission risk [1, 2], evidence-based control for the principle vector, *Aedes aegypti*, is still lacking [3, 4]. Disease transmission is driven by a number of factors including entomological and climatic variables [5, 6], but largely by prevailing herd immunity, population density and human movement [7, 8, 9]. Considering this, it remains important to describe how epidemics move through populations in space and time, as well as document those demographics most at risk.

Throughout 2014–16, two arboviruses, dengue (DENV) and Zika (ZIKV) were responsible for large disease outbreaks worldwide. Health systems were disrupted and resources exhausted amidst a huge mobilisation of labour and materials towards controlling the outbreaks. The Dominican Republic was similarly affected. In 2015/early-2016, a major dengue outbreak affected the island resulting in 17,324 cases in 2015 (18.0 per 10,000 population) and 6329 (6.6 per 10,000 population) in 2016. As this outbreak abated, the first cases of Zika were detected. Then by week 52 of 2016, 5,235 Zika patients (5.5 per 10,000 population), 22 cases of infant microcephaly and 600 cases of Guillain-Barre syndrome (GBS) had been reported.

Accordingly, using a retrospective disease surveillance dataset, this study sought to: 1) identify and describe the transmission dynamics of Zika and dengue across Dominican Republic including associated cases of Guillain-Barre syndrome (GBS), 2) compare the age and sex-adjusted distributions of both Zika and dengue outbreaks and 3) calculate the reproductive number (R0) of each outbreak.

## Methods

### Context

The estimated total population of Dominican Republic in 2015 was 9.98 million with the capital Santo Domingo accounting for around 27% or 2.65 million. In 2016, Santo Domingo received annual precipitation of 730mm with an average temperature of 27°C [10]. *Aedes* populations appear to be well established and contribute to endemic dengue transmission, although entomological surveys are unsystematically conducted, two to three times per year [11].

### Datasets

Data from years 2015–2016 were obtained from the Dominican Republic database, Sistema Nacional de Vigilencia Epidemiologica. Quality assurance of case reporting was conducted in a previous study [6] via monitoring visits while funds were made available for one additional monitoring visit to validate the quality of the data used in this study. The database was de-identified at source and epidemiological week was defined as Sunday through Saturday. There was no prior data cleaning/ manipulation of the raw dataset therefore adjustments for population age distributions were necessary. Variables included suspected, probable and confirmed Zika and dengue cases (according to WHO case definitions [12,13]), date of onset, date of notification, symptoms and other descriptive epidemiological/ demographic variables.

### Statistical analyses

Surveillance data were analysed using STATA 13.0 (STATA Corp, 2016), RStudio, version 0.99.903 [14] and Microsoft Excel [15]. Descriptive results comprised means, medians, interquartile ranges and proportions. Age categories were defined by decade unless otherwise specified. Where necessary, measures of frequency were age-standardised to produce adjusted metrics. Estimated national census data for dengue (2015), Zika (2016) and Guillain-Barre (2016) were used for all calculations. Pearson’s Chi-squared test for independence was used to compare odds of infection between categorical variables, namely sex and event.

Basic reproduction number (R0) calculations used the exponential growth method with Poisson regression and an assumed serial interval of 22 days (lognormal, standard deviation of 3) based on previous estimates [16]. In the absence of dengue seropositivity data and prior reported Zika cases, the human population was assumed to be naïve to both Zika and dengue for all R0 calculations.

### Ethics

Ethical clearance was granted by the Pan American Health Organization Ethics Review Committee (PAHO-ERC; Ref No. 2014-10-0023) and accepted by Dominican Republic Ministry of Health.

## Results

**Fig 1:**
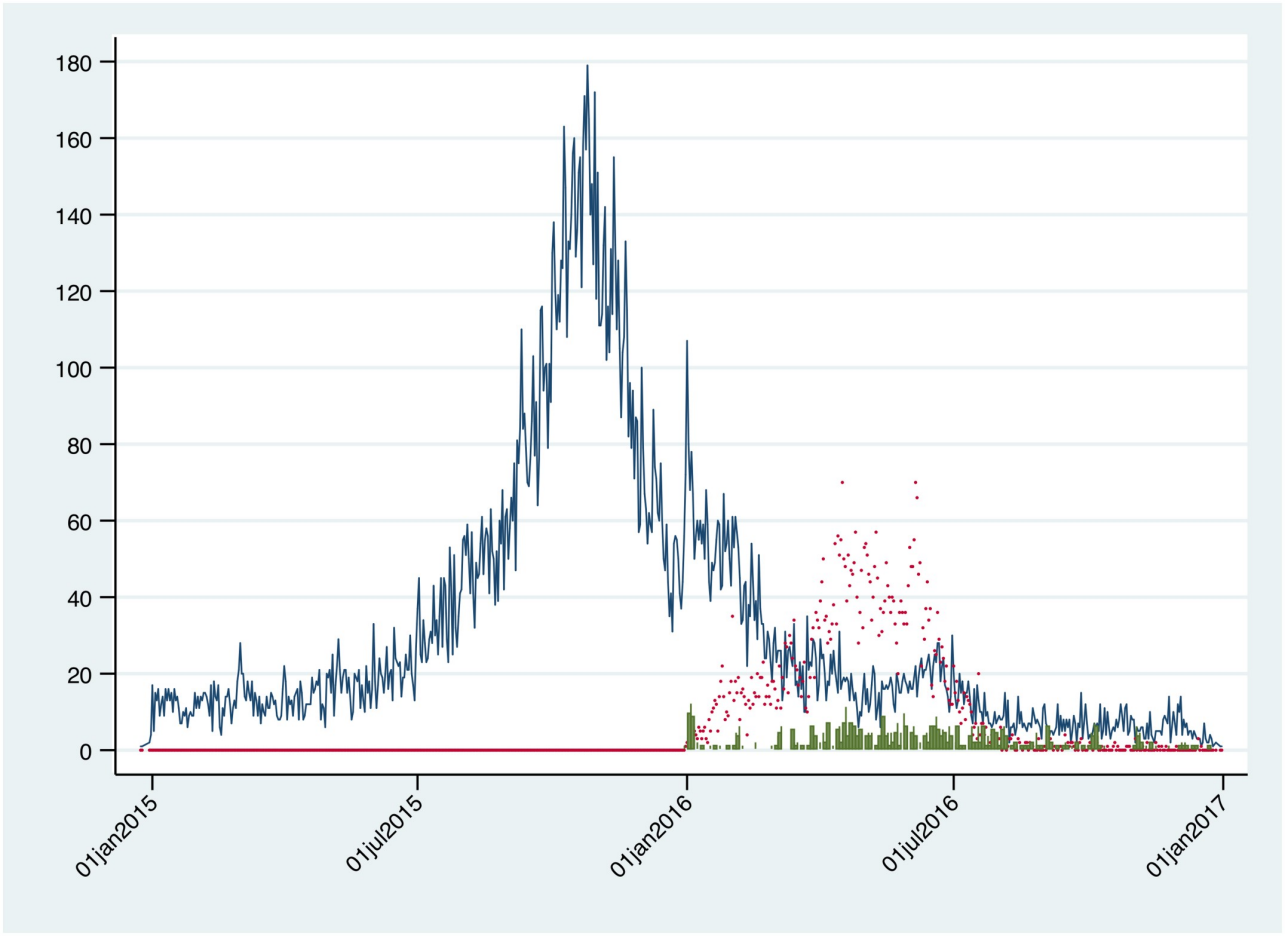
National time-series of daily reported incident cases of dengue, Zika and Guillaine-Barre syndrome cases from January 2015 –January 2017. Blue line: dengue. Red scatter: Zika. Green bar: Guillain-Barre syndrome.

Datasets were analysed to describe Zika, dengue and GBS nationally. Fig 1 shows a time-series plot of national suspected (WHO case definition) clinical infections of dengue, Zika and GBS by date of symptom onset. While dengue and Zika peaks are distinct, they are not independent in time. GBS incidence occurs simultaneously with the onset of Zika.

**Fig 2:**
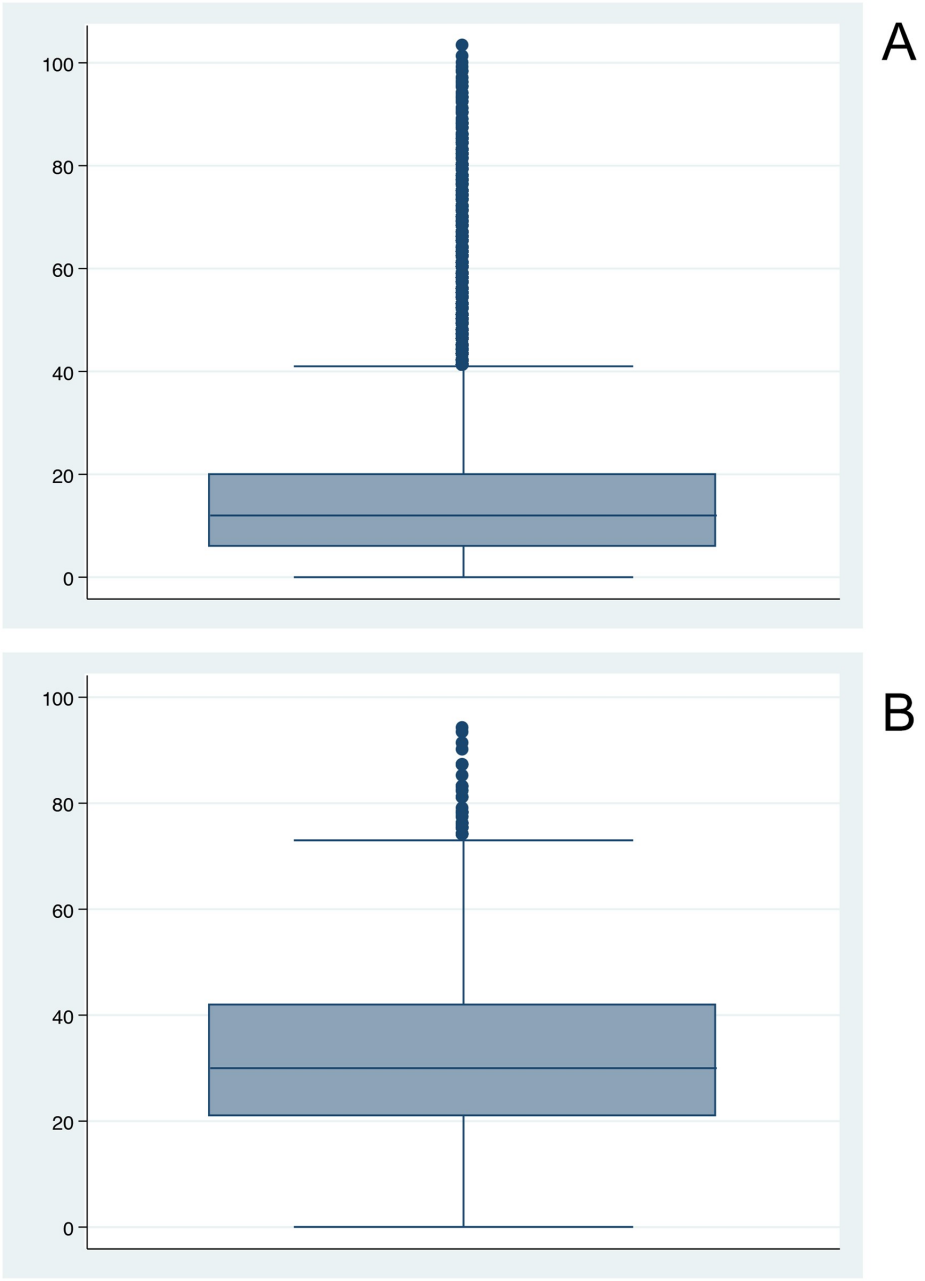
National distribution of suspected incident cases of dengue (2a) and Zika (2b).

**Fig 3:**
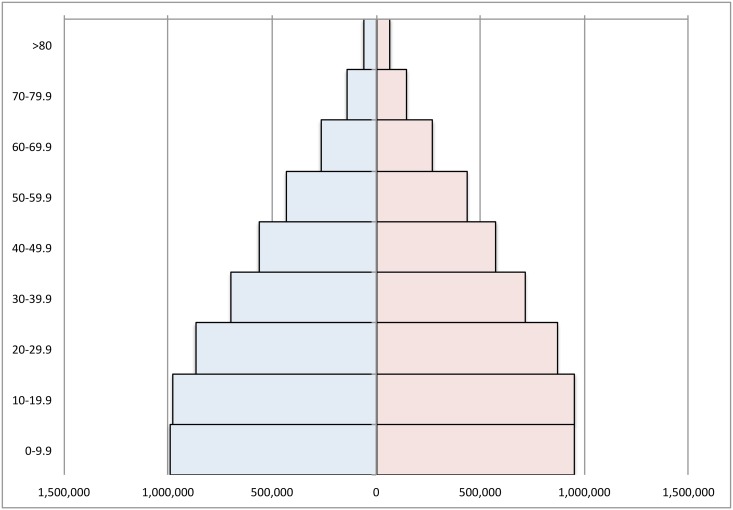
Population Pyramid of Dominican Republic by age and sex. Blue = males, red = females.

Boxplots established the distribution of cases among the population (Fig 2) and revealed that 75% of all dengue cases occurred before the age of 20 compared with ~42 for Zika. The population structure of Dominican Republic stratified by age can be seen in Fig 3.

**Fig 4:**
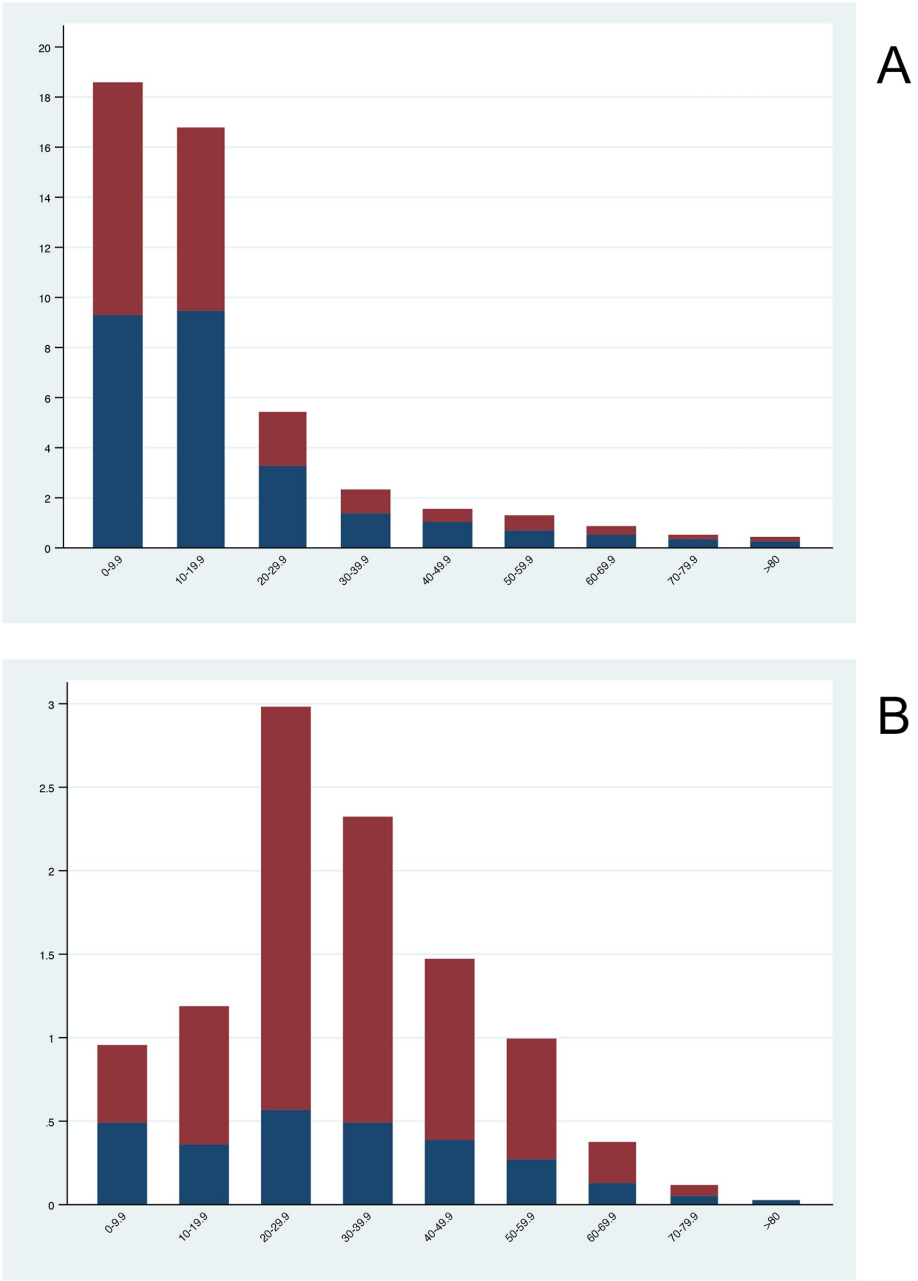
National sex-specific attack rates (per 10,000 population) stratified by age group where blue = male and red = female. Dengue (4a) and Zika (4b).

The national age and sex distribution of Zika and dengue cases can be observed in Fig 4. As a percentage, dengue infections were higher among men than women: 54.5% (95% Credible Interval (CI) 53.9%, 55.2%) vs. 45.5% (95% CI 44.7%, 46.0%) respectively. By comparison, Zika infection was markedly lower among men than women: 26.2% (95% CI 24.9%, 27.3%) vs. 73.8% (95% CI 72.6%, 75.0%) respectively. Pearson’s chi-squared test for independence was significant at p<0.0001 (unadjusted odds of dengue infection: 0.0026 vs. 0.0022, X^2^ = 198.79, p<0.0001 in males and females respectively; unadjusted odds of Zika infection: 0.00027 vs. 0.00077, X^2^ = 1200.00, p<0.0001 in males and females respectively). Crude and age-adjusted, sex stratified national attack rates can be observed in Table 1 below. Age-adjusted attack rates of dengue in males was 25.9 per 10,000 population vs. 21.5 per 10,000 population in females, while age-adjusted attack rates of Zika infection were 2.7 per 10,000 population in males vs. 7.6 per 10,000 population in females.

**Table 1:** Crude and age-adjusted national incidence per 10,000 population for dengue and Zika.

	Dengue attack rate (per 10,000 population)	Zika attack rate (per 10,000 population)	GBS (per 10,000 population)
**All Population**			
Crude	159.10	40.68	7.00
Adjusted	23.74	5.20	0.59
**Men**			
Crude	177.62	22.44	7.11
Adjusted	25.92	2.72	0.62
**Women**			
Crude	141.12	57.56	6.87
Adjusted	21.57	7.64	0.57

**Fig 5:**
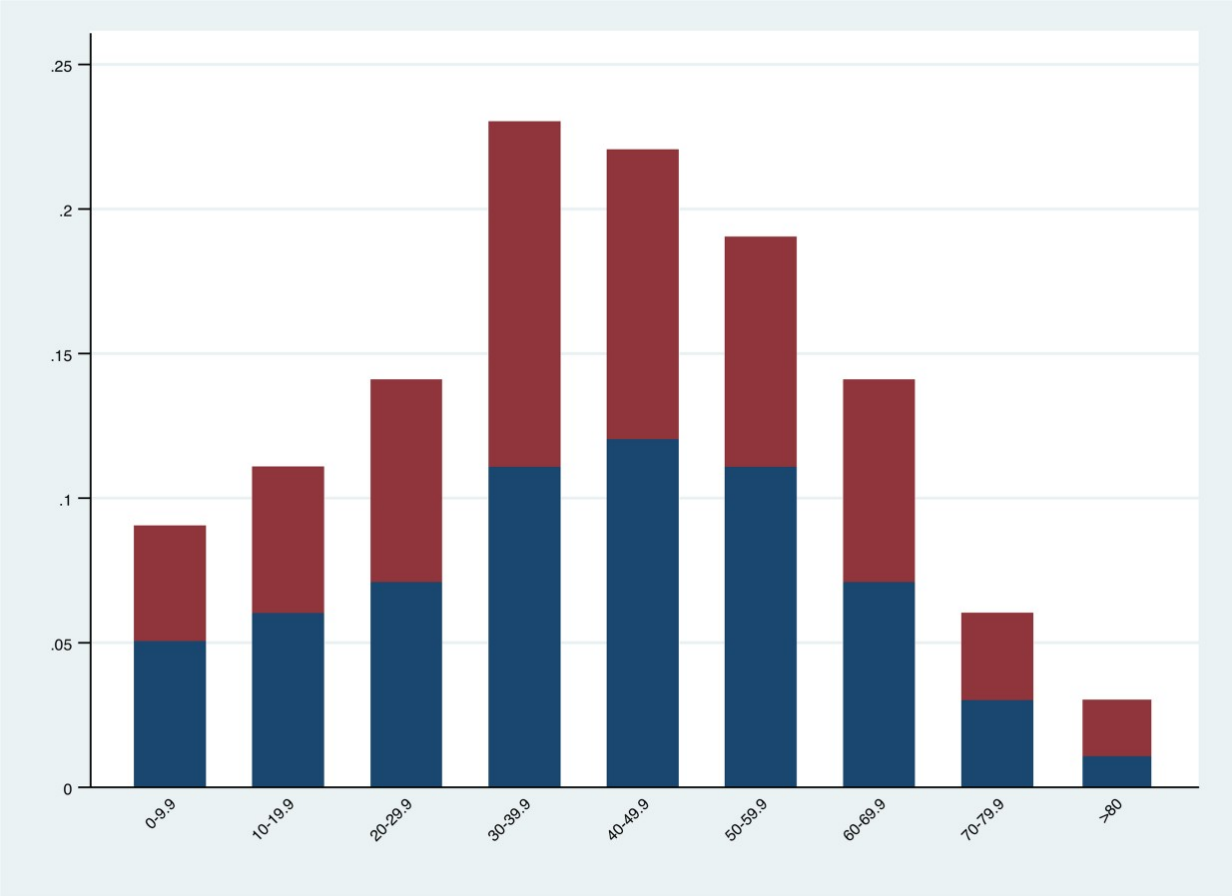
National sex-specific adjusted attack rates (per 10,000 population) for GBS stratified by age group where blue = male and red = female.

National, adjusted GBS attack rates were 0.59 per 10,000, with 0.62 and 0.57 per 10,000 among males and females respectively (Table 1). Across age groups, incidence was higher in those of reproductive age, with slightly higher incidence in males between the ages of 40 and 70 (Fig 5).

National R0 Zika calculations gave a national basic reproduction number of 1.80 (95% CI 1.78, 1.82). Stratifying by sex, the national R0 for males was 1.72 (95% CI 1.69, 1.75) and 1.84 (95% CI 1.82, 1.87) for females.

**Fig 6:**
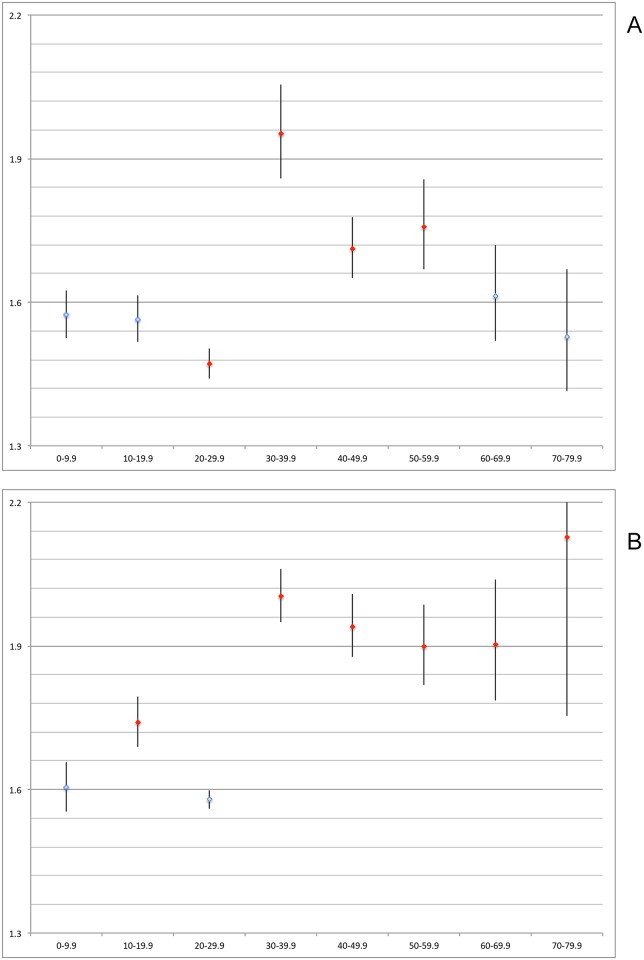
National age-stratified Zika R0 results with 95% credible intervals. Red markers indicate significant difference at p<0.05 when compared with age 0–9.9yrs. Y axis = R0; X axis = age group. Male: 6a. Female: 6b.

Age-stratified national R0 calculations demonstrated that males between 30-60yrs were significantly more likely to present with suspected Zika symptoms compared to baseline (p<0.05), while for females it was those between 10-20yrs and 30-80yrs who were more likely to present with suspected Zika (Table 2, Fig 6). In males and females between the ages of 20-30yrs Zika presentation was less likely although the difference was not statistically significant in females.

**Table 2:** R0 calculations stratified by age and sex with accompanying credible intervals (CI).

Age Group	Male R0 (CI)	Female R0 (CI)
0–9.9	1.57 (1,53, 1.62)	1.60 (1.55, 1.66)
10–19.9	1.56 (1.52, 1.62)	1.74 (1.68, 1.80)
20–29.9	1.47 (1.44, 1.50)	1.58 (1.56, 1.60)
30–39.9	1.95 (1.86, 2.06)	2.00 (1.95, 2.06)
40–49.9	1.71 (1.65, 1.78)	1.94 (1.88, 2.01)
50–59.9	1.76 (1.67, 1.86)	1.90 (1.82, 1.99)
60–69.9	1.61 (1.52, 1.72)	1.90 (1.79, 2.04)
70–79.9	1.53 (1.41, 1.67)	2.13 (1.75, 2.68)

## Discussion

During the time period July 2015—July 2016, Dominican Republic experienced two large flavivirus outbreaks as part of a continuing worldwide trend for increasing dengue and Zika incidence. This was followed by the onset of Zika-associated neurological and developmental conditions, known as Guillain-Barre syndrome and microcephaly. A retrospective analysis of the available data described the epidemiological characteristics of the outbreaks on a national level, stratified by age and sex, along with relevant adjustments for population structure, accompanied by crude R0 estimates to better describe the strength of transmission in the population.

That 75% of reported dengue infections occurred before the age of 20 is not unexpected, given the immune-naïve status of younger age groups in a country where dengue is endemic. It is equally unsurprising that a newly introduced virus, such as Zika, resulted in a much broader demographic of incident cases, due to a completely susceptible human population. Yet by contrast, when analyses were stratified by age, adjusted Zika attack rates were higher among 20-50yrs. This is likely due to a number of direct and indirect factors, namely: 1) higher mobility among younger demographics, leading to increased exposure to infected *Aedes* mosquitoes [7]; 2) sexual transmission among sexually-active age groups as a complementary driver of incidence [17]; 3) skewed impact of over-reporting among women of sexual reproductive age. These trends were also borne out among the GBS data although the effect was not nearly as pronounced.

Recent literature has documented cross-reactivity between dengue and Zika antibodies [18, 19, 20, 21]. In light of this, one might expect to observe a difference, in either direction, in the Zika incident rate in adults compared to infants and children, as much of the adult population in Dominican Republic is already dengue seropositive. This assumption is built on data indicating that prior dengue infection leads to both antibody-dependent enhancement (ADE) of Zika symptoms, as well as possible protective effects, such as the shortening of the Zika viraemic period [22]. And yet incident rates in this dataset are broadly similar between the susceptible and naïve demographics: Zika attack rates among males and females between 50-60yrs (and males between 40-50yrs) were similar to infants and children. This suggests that while cross-reactivity occurs at the molecular level, this might not be sufficient to prevent or enhance symptomatic Zika disease at the population level [23]. While disease severity was not an outcome of this study, cross-reactivity may be either limiting or indeed accelerating associated Zika morbidity or mortality among dengue-seropositive populations [24], but as yet remains an outstanding knowledge gap. Also, it is not known whether congenital antibodies confer protection against microcephaly [24].

As has been noted elsewhere [25], males were at greater risk of dengue infection. This could be due to increased mobility, workplace exposure [26] and/or increased health-seeking behaviour [27]. Interestingly, sex-specific Zika attack rates were positively skewed towards females. Indeed 74% of total cases were among females with a trend towards a greater burden among those of sexual reproductive age. The evidence is therefore suggestive of increased female susceptibility to Zika infection, as has been noted in recent literature [17]. However this could be confounded by both increased health-seeking behaviour among this risk group as well as the presence of pregnant women among our data. And yet similar results are prominent in other articles [28, 16). To better understand this trend, we used GBS as a possible proxy to represent true Zika infection distribution between the sexes. The data conversely showed that adjusted, stratified GBS attack rates were skewed towards males, which is counter to the trend one might expect were females more susceptible to Zika infection. Yet upon examination of global GBS rates in a systematic review and meta-analysis, Sejvar et al., (2011) [29] demonstrated that attack rates are on average higher (almost double) in males than females, which persists and steadily rises with age [29]. In relation to the data seen here, the gap between male and female rates is narrower than expected, which might indicate absolute increased infection of Zika among women in Dominican Republic, but one should caution that the data from Sejvar et al., 2011 [29] were modelled, which prevents overarching parallels being drawn. Considering the data and variety of plausible confounders, true associations to explain the higher incident rate among females remain evasive. While there might still be a biological cause, it is more likely that successful, targeted media campaigns are influencing the health seeking behaviour of women of reproductive age, in turn leading to over-reporting of Zika infections among women.

The R0 is used widely to establish the strength or intensity of a given outbreak, where >1 results in continuing spread and <1 results in suppression of disease transmission. However any model estimations can change based on the parameterisation of the model used [30] and can be limited by surveillance bias, in this case, possible sex-specific over-reporting. Data are likely further confounded by reporting biases, including a large ratio of asymptomatic to symptomatic patients [30], as well as likely under-reporting among specific demographics, such as the MSM (men-who-have-sex-with-men) community [17]. Indeed these limitations are borne out by the data presented in this article, where although R0 was significantly higher in women thereby reflecting the incident risk calculations, there remained a consistent inexplicable artefact for ages 20-30yrs, which showed lower strength of transmission relative to baseline (albeit statistically insignificant among females). This is likely due to spatio-temporal effects within this highly mobile age group. Additionally, these crude estimates were not adjusted for age/sex distributions, although still they largely reflected sex-specific incident rates: R0 was significantly higher in all age groups of increasing mobility and reproductive age, relative to 0-10yrs. Together, the R0 trend suggests faster Zika transmission among highly mobile, sexually active females but as previously noted, this is likely due to health-seeking behaviour. Considering this, the headline Zika R0 of 1.80 and associated sex/age specific attack rates should be taken with caution while the overuse of the R0, particularly when attempting to establish trends across age and sex, should be discouraged.

These data and resulting calculations demonstrate both the strengths and weaknesses of epidemiological metrics. Importantly, it is clear that descriptive and analytical approaches should be both well-considered and flexible to afford the time and resources necessary for the exploration of data trends and scientific hypotheses to ensure a balanced approach to modelled data.

### Limitations

National surveillance data, especially the use of suspected cases, will be subject to some misclassification of disease (Zika as dengue) during the early stages of the outbreak. Categorisation by age will have resulted in the presence of residual confounding that may marginally influence the results. R0 calculations assumed that the human population was naïve to both Zika and dengue, which may have resulted in a negative bias if existing herd immunity to either infection was high. Additionally, results between ages 70-80yrs remain unreliable due to large credible intervals while R0 calculations for 80+yrs could not be completed due to paucity of data. Notably, it was not possible to remove pregnant women from the dataset to control for media campaigns targeting this demographic. Finally, the R0 artefact within the 20-30yr-age band remains inexplicable.

### Recommendations

Results indicate that antibody cross-reactivity may not be influencing reported outcomes at the population level, although disease severity was not an outcome in this study. Evidence also suggests a greater burden of Zika among women, however this is most likely due to increased health-seeking behaviour rather than biological mechanisms, yet the latter still cannot be ruled out. If correct, targeted advocacy campaigns are effective at changing short-term behaviour and are recommended during Zika epidemics.

Confounding of incident risk and R0 calculations by impactful advocacy campaigns can alter the reliability of epidemiological metrics, which demonstrates the limitation of using descriptive statistics alone to describe the passage of outbreaks. The use of associated proxy syndromes such as Guillain-Barre syndrome to mitigate confounding of passive surveillance data by targeted health campaigns may be of limited use.
